# HPVTIMER: A shiny web application for tumor immune estimation in human papillomavirus‐associated cancers

**DOI:** 10.1002/imt2.130

**Published:** 2023-08-12

**Authors:** Liying Liu, Yanan Xie, Hong Yang, Anqi Lin, Minjun Dong, Haitao Wang, Cangang Zhang, Zaoqu Liu, Quan Cheng, Jian Zhang, Shuofeng Yuan, Peng Luo

**Affiliations:** ^1^ Department of Oncology Zhujiang Hospital, Southern Medical University Guangzhou Guangdong China; ^2^ The First Clinical Medical School Southern Medical University Guangzhou Guangdong China; ^3^ The Second Clinical Medicine School Southern Medical University Guangzhou Guangdong China; ^4^ Department of Surgical Oncology Sir Run Run Shaw Hospital Affiliated to Zhejiang University, School of Medicine Hang Zhou China; ^5^ Thoracic Surgery Branch Center for Cancer Research, National Institutes of Health Bethesda Maryland USA; ^6^ Department of Pathogenic Microbiology and Immunology Xi'an Jiaotong University Xi'an Shaanxi China; ^7^ Department of Interventional Radiology The First Affiliated Hospital of Zhengzhou University Zhengzhou Henan China; ^8^ Department of Neurosurgery Xiangya Hospital, Central South University Changsha Hunan China; ^9^ National Clinical Research Center for Geriatric Disorders, Xiangya Hospital Central South University Hunan China; ^10^ Department of Infectious Disease and Microbiology The University of Hong Kong‐Shenzhen Hospital Shenzhen China; ^11^ State Key Laboratory of Emerging Infectious Diseases, Department of Microbiology School of Clinical Medicine, Carol Yu Centre for Infection, Li Ka Shing Faculty of Medicine, The University of Hong Kong Hong Kong China

**Keywords:** biomarkers, HPV‐associated cancers, HPVTIMER, tumor immune microenvironment, web tools

## Abstract

The tumor immune microenvironment (TIME) is closely associated with tumor formation, particularly linked to the human papillomavirus (HPV), and regulates tumor initiation, proliferation, infiltration, and metastasis. With the rise of immunotherapy, an increasing amount of sample data used for TIME exploration is available in databases. However, no currently available web tool enables a comprehensive exploration of the TIME of HPV‐associated cancers by leveraging these data. We have developed a web tool called HPV‐associated Tumor Immune MicroEnvironment ExploreR (HPVTIMER), which provides a comprehensive analysis platform that integrates over 10,000 genes and 2290 tumor samples from 65 transcriptome data sets across 8 cancer types sourced from the Gene Expression Omnibus (GEO) database. The tool features four built‐in analysis modules, namely, the differential expression analysis module, correlation analysis module, immune infiltration analysis module, and pathway analysis module. These modules enable users to perform systematic and vertical analyses. We used several analytical modules in HPVTIMER to briefly explore the role of *CDKN2A* in head and neck squamous cell carcinomas. We expect that HPVTIMER will help users explore the immune microenvironment of HPV‐associated cancers and uncover potential immune regulatory mechanisms and immunotherapeutic targets. HPVTIMER is available at http://www.hpvtimer.com/.

## Highlights

- HPVTIMER provides multiple analysis modules for exploring differences between HPV‐positive and HPV‐negative samples, based on transcriptome data, such as differential expression analysis, correlation analysis, immune infiltration analysis, and pathway enrichment analysis.
- The immune infiltration analysis module provides users with seven specific algorithms, including CIBERSORT, CIBERSORT absolute mode, MCP‐counter (or mMCP‐counter for mice), EPIC, xCell, quanTIseq, and TIMER.
- HPVTIMER provides users with meta‐analysis, which can be used to explore correlations between gene expression/pathway single‐sample gene set enrichment analysis (ssGSEA) scores/immune infiltration scores and differences in immune infiltration scores between HPV‐positive/negative samples.

## Graphical abstract

HPV‐associated Tumor Immune MicroEnvironment ExploreR (HPVTIMER) integrates transcriptomic data from the National Center for Biotechnology Information (NCBI) Gene Expression Omnibus (GEO) database for HPV‐associated cancers to allow users to perform various analyses, including differential expression analysis, correlation analysis, pathway analysis, and immune infiltration analysis based on HPV infection status.

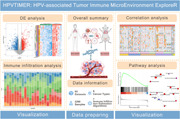

## INTRODUCTION

Human papillomavirus (HPV)‐associated cancers refer to cancers that are associated with high‐risk HPV infections occurring in various sites but share similar biological and immunological properties [1]. These cancers are thought to have unique viral antigens that can be specifically targeted by novel immunotherapies such as immune checkpoint inhibitors, adoptive cell therapies, and therapeutic vaccines. Despite numerous clinical investigations, the response rates to these therapies observed to date have been modest, and resistance has emerged in some cases [1, 2, 3]. This finding may be attributed to the lack of understanding of the immunomodulatory mechanisms of HPV‐associated cancers and the varied immunological characteristics of different primary sites [1, 4]. Meanwhile, an increasing number of studies have demonstrated that the unique tumor immune microenvironment (TIME) induced by HPV may play a crucial role in tumor development [5, 6, 7]. Some recent studies have revealed that HPV blocks the activation of cytotoxic T cells and NK cells by affecting the antigen presentation pathway [1, 8]. Despite the presence of a high number of CD8+ T cells in the stroma of cervical carcinomas (CC), these cells are ineffective in inhibiting tumor growth due to the HPV‐induced downregulation of chemoattractants (e.g., CCL20) which limits the trafficking of antigen‐presenting cells such as Langerhans cells to the site of infection [8]. In addition, some evidence shows a high number of FOXP3+ regulatory T cells (Tregs) in both CC and head and neck squamous cell carcinomas (HNSCC) [1, 5, 9, 10, 11, 12], which suppress antitumor immunity by downregulating the induction and proliferation of effector T cells. The abundance of Treg cells in cervical cancer has been associated with disease severity [13, 14], and whether Treg cells are more prevalent in HPV‐positive HNSCC than in HPV‐negative HNSCC is controversial [5, 9, 10, 11, 12, 15, 16]. Therefore, a more comprehensive exploration of the TIME of HPV‐associated cancers is crucial to uncover immunomodulatory mechanisms and facilitate the development of more effective immunotherapeutic strategies.

Given the common causality of high‐risk HPV infection, the immune microenvironments of HPV‐associated cancers share similarities. However, it is important to recognize that the various primary sites of HPV‐associated cancers exhibit distinct clinical behaviors, which may reflect differences in the density and distribution of immune cells in the TIME that vary by cancer type [17]. Therefore, elucidation of the distinct molecular and immune profiles that differentiate HPV‐positive and HPV‐negative tumors across different primary sites of HPV‐associated cancers is crucial to facilitate the development of personalized and precise immunotherapeutic strategies. In recent years, immunotherapy for HPV‐associated cancers and advancements in sequencing technologies have increased the interest in exploring the TIME of HPV‐associated cancers. Although a vast amount of transcriptomic data of HPV‐associated cancers is stored in public databases, it can be challenging for users without expertise in immune infiltration analysis to extract relevant information. To address this issue, a web‐based tool that aggregates information on the TIME of multiple HPV‐associated cancers and thus allows users to perform comparative analysis would greatly benefit basic researchers and clinicians. Currently, several web tools, such as TIMER2.0 [17], TIP [18], and CancerImmunityQTL [19], are available for tumor immune infiltration analysis. Although these web tools are valuable and simultaneously encompass two major HPV‐associated cancers (cervical cancer and head and neck cancer), they are not specifically designed for HPV‐associated cancers and do not emphasize the impact of HPV infection on the TIME. Additionally, databases and analysis tools, such as HNCDB [20], HNOCDB [21], HNdb [22], OrCGDB [23], and CCDB [24], exist for specific cancer types, even though they do not include information on most common types of HPV‐associated cancers. Moreover, these tools do not perform immune infiltration analysis. Furthermore, except for HNCDB, these resources do not offer a comprehensive understanding of the influence of HPV infection on the biological characteristics of HPV‐associated cancers.

In conclusion, the current web‐based tools for studying HPV‐associated cancers are lacking in comprehensiveness and functionality. To address this issue, a new platform named HPV‐associated Tumor Immune MicroEnvironment ExploreR (HPVTIMER) was developed. This platform integrates transcriptomic data from the National Center for Biotechnology Information (NCBI) Gene Expression Omnibus(GEO) database for HPV‐associated cancers to allow users to perform various analyses, including differential expression analysis, correlation analysis, pathway analysis, and immune infiltration analysis based on the HPV infection status. HPVTIMER features multiple credible algorithms for immune infiltration analysis and horizontal analysis and the results are presented in aesthetic charts that can be easily downloaded. The goals of HPVTIMER are to facilitate the exploration of the molecular and immune landscapes of HPV‐associated cancers across different primary sites and to uncover differences between HPV‐positive and HPV‐negative tumors.

## RESULTS

**Figure 1:**
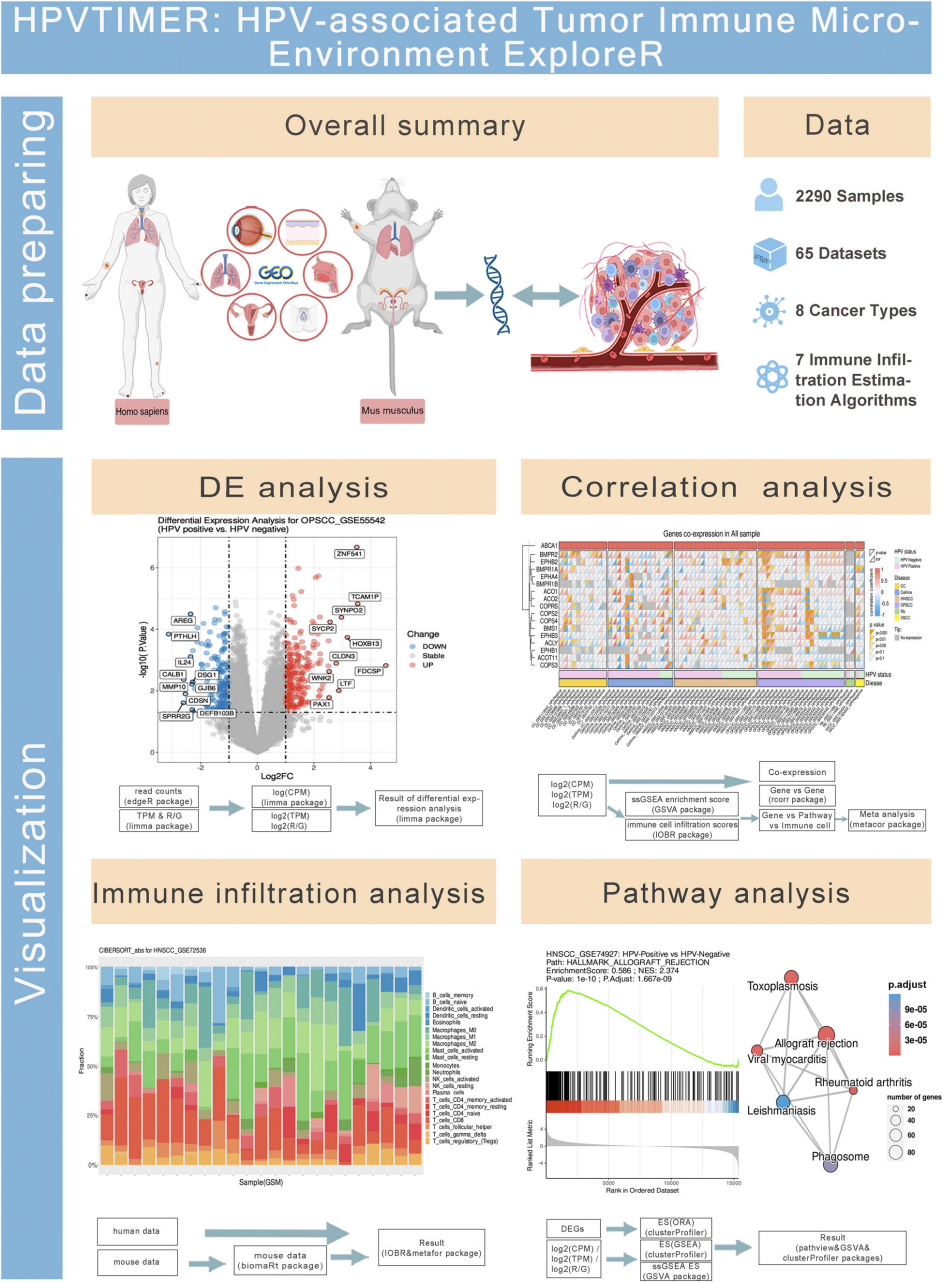
Flowchart of HPVTIMER. The upper panel shows the data preparation part and the lower panel shows the four main data analysis modules. The detailed steps for each analysis are also provided in the diagram.

HPVTIMER comprises 65 transcriptomic data sets of HPV‐associated tumors, covering two species (human and mouse) and eight cancer species. The transcriptome data were preprocessed and standardized based on their kind. HPVTIMER provides users with four analysis modules, namely, the differential expression analysis module, correlation analysis module, immune infiltration analysis module, and pathway analysis module (Figure 1). These four analysis modules enable users to investigate the properties of signatures of interest in HPV‐associated tumor HPV‐positive/negative samples.

### Differential expression (DE) analysis

**Figure 2:**
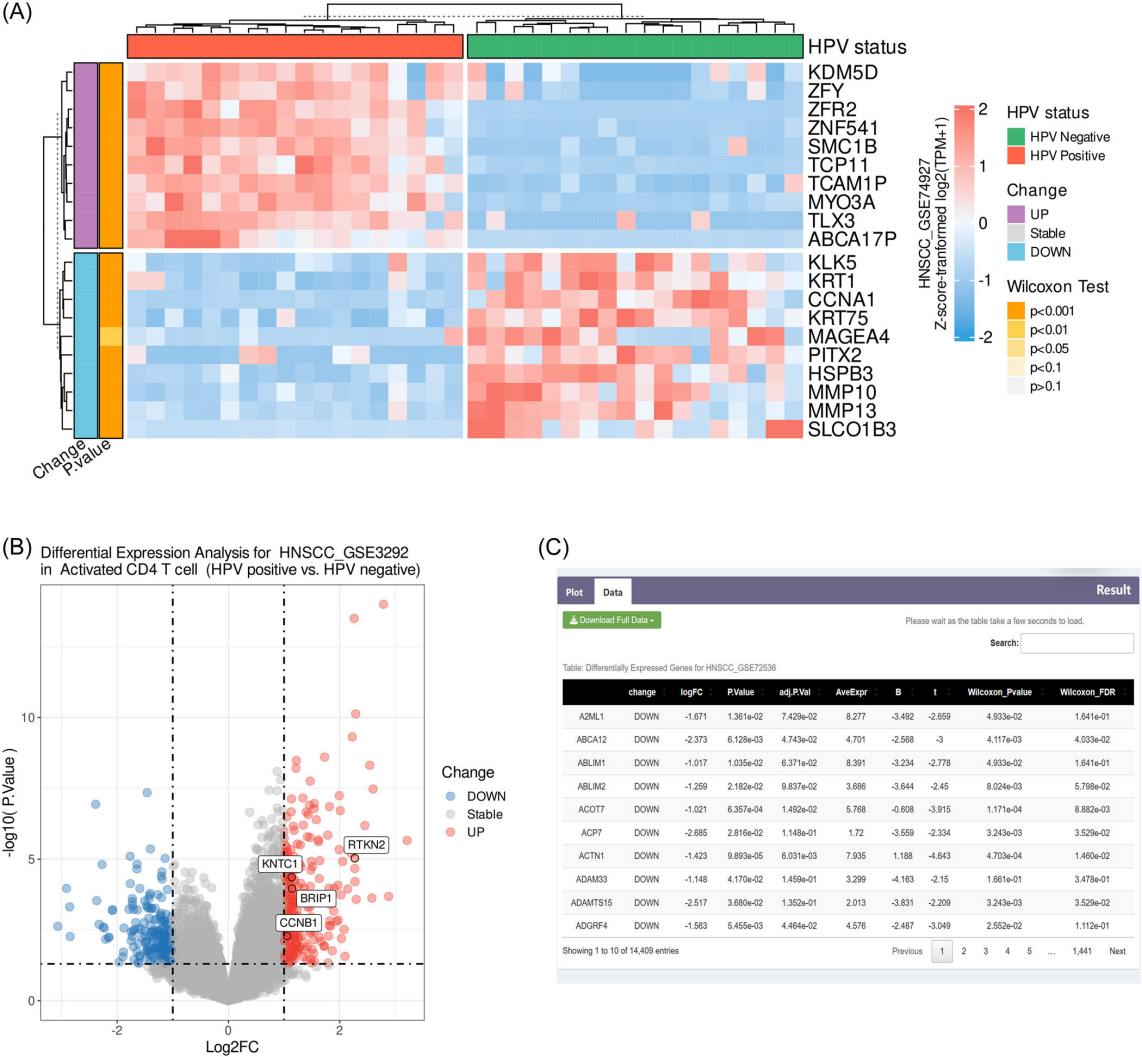
Visualization of DE analysis. (A) Heatmap of the expression of the genes of interest in the data set. The Wilcoxon–Mann–Whitney test was used to compare whether the genes of interest differ between HPV‐positive and HPV‐negative samples. (B) Differential expression volcano plot of the data set of interest. The genes of interest are labeled in text boxes. (C) Summarizing table of the results from DE analysis. Results of the DE analysis were obtained using the limma package. Specifically, “Wilcoxon_Pvalue” and “Wilcoxon_FDR” are additionally calculated by the Wilcoxon–Mann–Whitney test. DE, differential expression.

DE analysis module allows the user to perform DE analysis to identify genes that are differentially expressed between cancer samples of the two HPV statuses (positive/negative). It includes three submodules, namely, overall, immune cells, and pathways. The results are visualized as heatmaps (Figure 2A), volcano maps (Figure 2B), and box plots (Figure S1), and users can change the color scheme of the graph using “Customize.” In the heatmaps and box plots, the Wilcoxon–Mann–Whitney test is used to determine whether the presented genes showed significant differential expression between HPV‐positive and HPV‐negative samples. Under the Data tab, users can view the results of the differential expression profiling of the data set (Figure 2C).

#### Overall

Users can obtain the DE analysis results by selecting the data set of interest. Using “Customize,” users can set the fold change and significance thresholds and choose to visualize custom genes or the most up‐ or down‐regulated genes sorted by the |log_2_FC| values.

#### Immune cells

This module allows users to visualize differentially expressed genes related to the immune cells of interest. The list of immune cell‐associated genes used in this module was obtained from Charoentong et al. [25], Rooney et al. [26], and Vésteinn Thorsson et al. [27] (Table S1). We only support the demonstration of significantly differentially expressed genes related to immune cell‐related genes.

#### Pathways

This module allows users to visualize differentially expressed genes related to the pathway of interest. We collected a total of 17,970 pathway‐associated genes from hallmark gene sets, canonical pathways, and ontology gene sets from the molecular signatures database (MSigDB) [28]. We only support the visualization of significantly differentially expressed genes among the pathway‐related genes.

### Correlation analysis

The correlation analysis module allows users to explore the correlation between gene expression, immune cell infiltration fraction, and single‐sample gene set enrichment analysis (ssGSEA) scores. It includes three submodules, namely, the co‐expression, multigenes, scatter plot, and meta‐analysis.

#### Co‐expression

**Figure 3:**
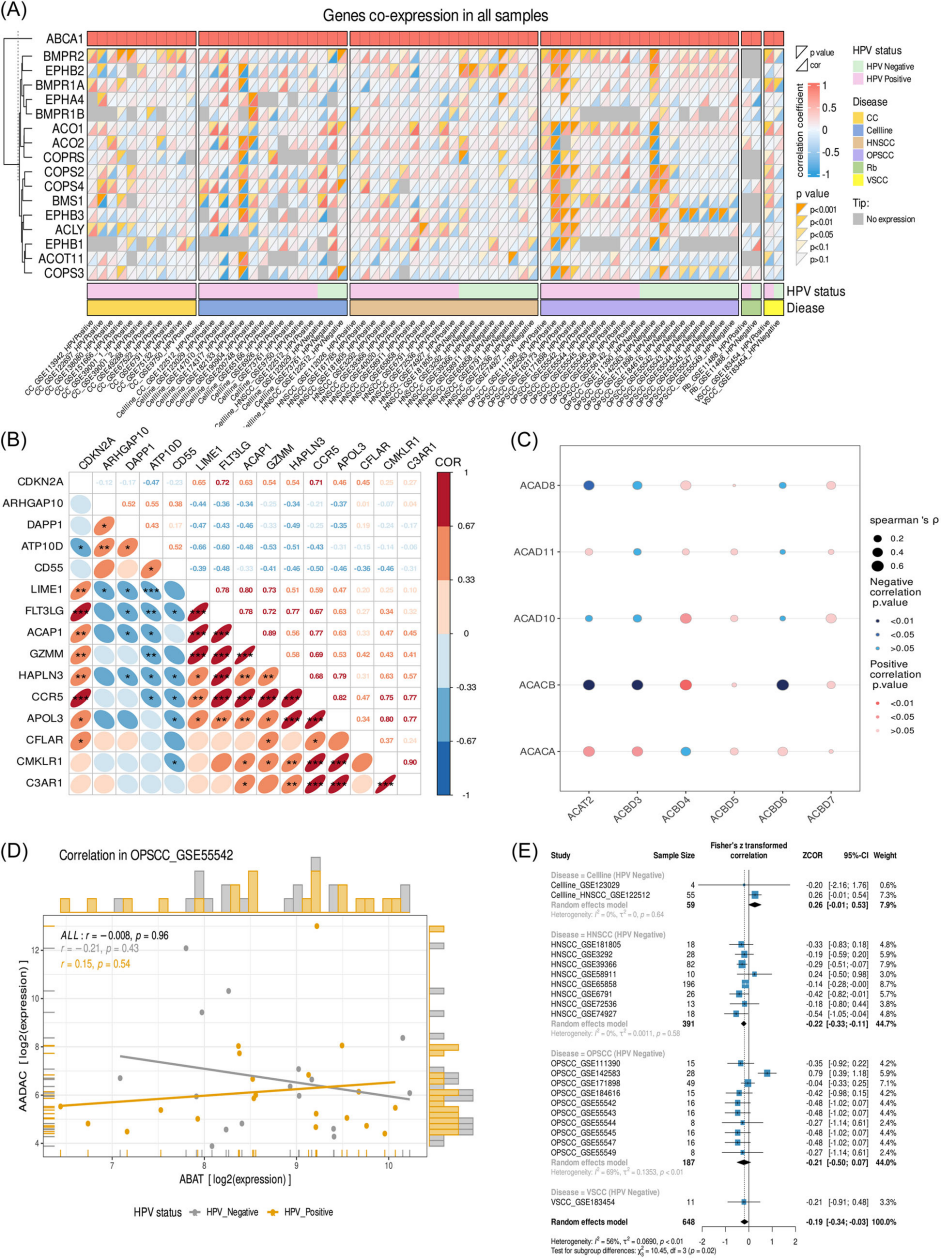
Example of correlation analysis. (A) Heatmap of co‐expression analysis. Each column of the heatmap represents a data set, and the color shades of each cell reflects the correlation coefficient and significance between the selected gene and the key gene, where the lower triangle indicates the correlation coefficient and the upper triangle indicates the corresponding significance. (B) Heatmap of correlation matrix coefficients. The values marked on the upper right of the graph are the correlation coefficients of the corresponding genes. *p < 0.05; **p < 0.01; ***p < 0.001. (C) Correlation coefficient bubble matrix. The size of the bubbles corresponds to the absolute value of the correlation coefficient. The red bubbles indicate positive correlations, and the blue bubbles indicate negative correlations. The color intensity of the bubbles indicates the absolute value of the *p* value. (D) The scatter plot shows the results of correlation analysis between the expression of the two genes of interest. (E) The forest plot shows the correlation between the gene expression/pathway ssGSEA scores/immune cell infiltration scores in multiple data sets. Within the example, a total of 22 data sets were included for analysis, involving three cancer types, HNSCC, OPSCC, and VSCC. HNSCC, head and neck squamous cell carcinomas; OPSCC, oropharyngeal squamous cell carcinoma; VSCC, vulvar squamous cell carcinoma.

This module allows users to explore correlations between the target genes and genes of interest within the overall sample across all samples. Users can select the species, target gene, sample range (all samples/HPV‐positive samples/HPV‐negative samples), correlation analysis method (Pearson's or Spearman's correlation analysis), and genes of interest. The results are visualized as heatmaps (Figure 3A).

#### Multigenes

This module allows users to explore the correlation between two lists of genes of interest. Users can select the correlation analysis method (Pearson's or Spearman's correlation analysis) and sample range (all samples/HPV‐positive samples/HPV‐negative samples). The correlation matrix of this module is visualized as heatmaps (Figure 3B) and bubble matrices (Figure 3C).

#### Scatter plot

This module allows users to perform a correlation analysis of the gene expression levels/ssGSEA scores/immune cell infiltration scores in the data set of interest. The user can choose the correlation analysis method (Pearson's or Spearman's correlation analysis) and the sample range (all samples/HPV‐positive samples/HPV‐negative samples). The results are visualized as scatter plots (Figure 3D).

#### Meta‐analysis

This module performs a meta‐analysis of the correlation coefficients between the gene expression/pathway ssGSEA scores/immune cell infiltration scores based on all data sets. Users can select the species (“Homo sapiens” or “Mus musculus”), cancer species, correlation analysis method (Pearson or Spearman), and sample range (all samples/HPV‐positive samples/HPV‐negative samples). Using “Customize,” users can adjust the meta‐analysis by selecting the meta‐analysis model, summary measure, and whether to perform subgroup analysis based on cancer species. The results are visualized as forest plots (Figure 3E).

### Immune infiltration

The immune infiltration analysis module allows users to explore the infiltration level of each immune cell in the tumor microenvironment using seven immune infiltration estimation algorithms (CIBERSORT, CIBERSORT absolute mode, MCP‐counter (or mMCP‐counter for mice), EPIC, xCell, quanTIseq, and TIMER). It includes two submodules, immune cells, and meta‐analysis.

#### Immune cells

**Figure 4:**
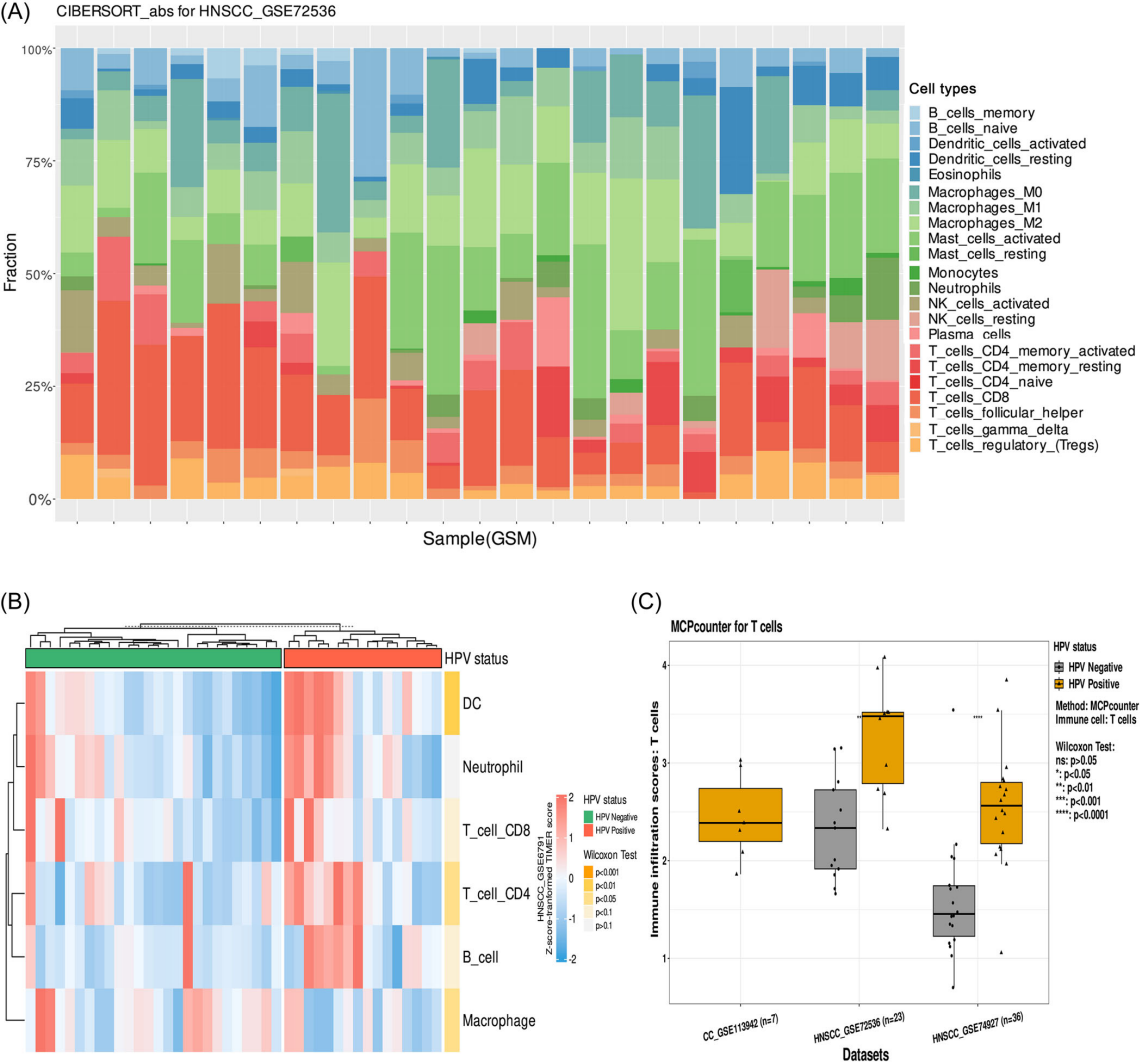
Example of immune infiltration analysis. (A) Stacked bar plot showing the relative proportion of tumor‐infiltrating immune cells. (B) Heatmap of infiltration scores for the data set of interest. The results of the analysis based on the Wilcoxon–Mann–Whitney test comparing HPV‐positive and HPV‐negative samples are labeled on the far right. (C) Presentation of the infiltration scores of the immune cells of interest for multiple data sets. The Wilcoxon–Mann–Whitney test was used to compare infiltration scores between different groups within the same data set. ns *p* > 0.05; *p < 0.05; **p < 0.01; ***p < 0.001; ****p < 0.0001.

Users can select the data set of interest and immune infiltration method of interest. The results are presented as stacked bar plots (Figure 4A), heatmaps (Figure 4B), and box plots (Figure 4C). In the box plots, the Wilcoxon–Mann–Whitney test is used to determine the significance of the difference in immune cell immune infiltration scores between HPV‐positive and HPV‐negative samples.

#### Meta‐analysis

This module performs a meta‐analysis of immune cell infiltration scores to explore the difference between HPV‐positive and HPV‐negative samples. Users can select the immune infiltration algorithm, and immune cells for meta‐analysis. Using “Customize,” users can adjust the meta‐analysis by selecting the meta‐analysis model, summary measure, and whether to perform subgroup analysis based on cancer species. The results are visualized as forest plots.

### Pathway analysis

The pathway analysis module allows users to use three enrichment algorithms (overrepresentation analysis [ORA], gene set enrichment analysis [GSEA], and ssGSEA) to explore certain gene functional features and biological processes associated with the HPV infection status of a tumor. It includes three submodules, pathway enrichment, pathview, and ssGSEA.

#### Pathway enrichment

**Figure 5:**
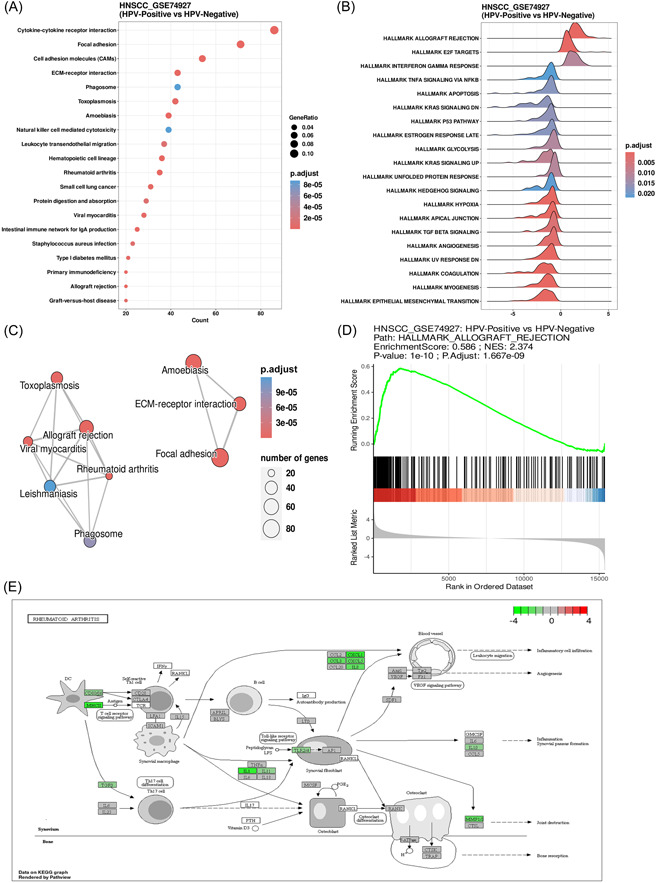
Example of pathway analysis. The pathway enrichment analysis shows the upregulation and downregulation of specific pathways in dot plots (A), ridgeline plots (B), Emap plots (C), and GSEA plots (D). (E) The pathway plot depicts gene expression changes in the kyoto encyclopedia of genes and genomes (KEGG) rheumatoid arthritis pathway between HPV‐positive and HPV‐negative samples in GSE183454. Green indicates downregulation, and red indicates upregulation.

Users can select the enrichment algorithm of interest (ORA or GSEA), the data set of interest, and the pathway set of interest to perform pathway enrichment analysis. Users can choose to display the top 20 enriched pathways or the pathways of interest. The results are presented as dot plots (Figure 5A), ridgeline plots (Figure 5B), enrichment maps (Figure 5C), GSEA plots (Figure 5D), and bar plots (Figure S2A).

#### Pathview

This module allows users to view kyoto encyclopedia of genes and genomes (KEGG) pathway maps. We provide users with significantly enriched KEGG pathways identified by the ORA and GSEA algorithms in the pathway enrichment module. In the pathway plot (Figure 5E), genes are colored according to their rank in the genome: red indicates a high degree of upregulation, and green indicates a low degree of downregulation.

#### ssGSEA

The ssGSEA module allows users to visualize the results of ssGSEA for multiple pathways of interest. The results are presented as heatmaps (Figure S2B).

### Data

The data module allows users to view detailed information on 65 data sets. It includes three submodules, data sets, clinical data, and immune cells.

#### Data sets

This module presents specific information on the 65 data sets embedded in the HPVTIMER website (Table S2). Users can filter the data sets of interest using the three selection boxes at the top of the page. The first table provides basic information such as the species, cancer type, platform, and study design (Figure S3A). When users select a data set on the first table, more specific information about the data set is shown in the second table, and this information includes whether the analysis can be performed for each module and the types of data, and so forth (Figure S3A). Users can download the complete data set summary table by clicking the download button in the upper right corner.

#### Clinical data

This module presents clinical characteristics of the 30 data sets that can be obtained and analyses for differences in clinical characteristics based on the HPV infection status.

#### Immune cells

This module shows the list of immune cell‐related genes used in the immune cells module of DE analysis (Figure S3B, Table S1) and allows users to search for immune cells, gene lists, and literature sources. Users can download the full table by clicking the download button at the bottom of the page.

### Candidate gene screening

**Figure 6:**
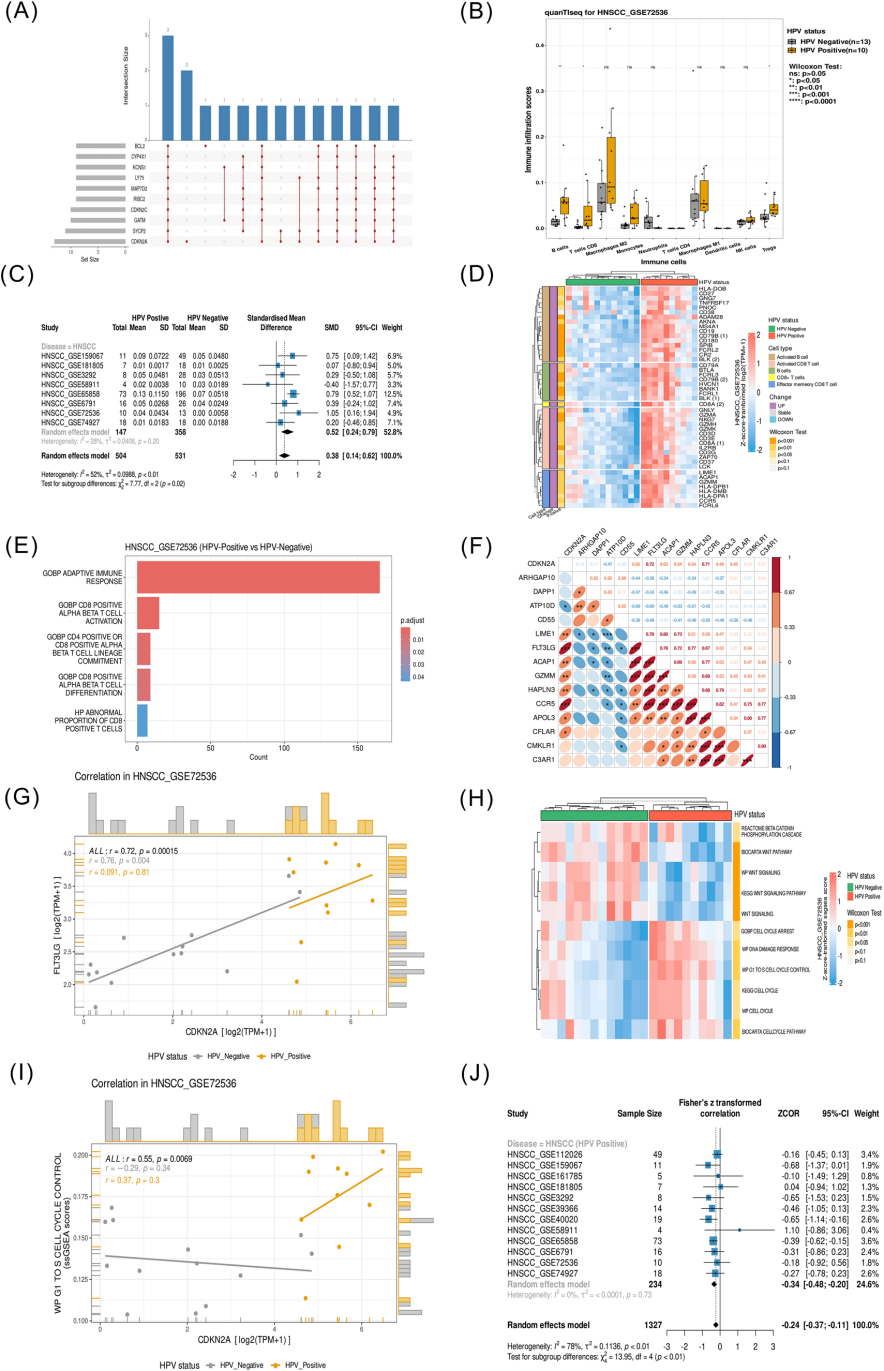
*CDKN2A* as a marker of HPV‐related cancers and immune infiltration analysis. (A) Upset plot shows the top 10 most significantly upregulated genes in all 65 built‐in data sets. Bar‐plot at bottom left indicates how many data sets the gene appears in as a significantly upregulated gene. The top bar‐plot represents the intersection size, and the linked point below indicates the selected gene. The figure suggests that *CDKN2A* is significantly upregulated in 13 data sets with the highest frequency; the 10 genes shown in the figure are simultaneously significantly upregulated genes in 3 data sets. (B) Comparison of immune infiltration scores (based on the quanTIseq algorithm) between HPV‐positive and HPV‐negative samples in HNSCC_GSE72536 using the Wilcoxon–Mann–Whitney test. ns: *p* > 0.05; *p < 0.05; **p < 0.01; ***p < 0.001; ****p < 0.0001. (C) The forest plot demonstrates the variability of CD8+ T cells' immune infiltration scores (based on the quanTIseq algorithm) between HPV‐positive and HPV‐negative samples. Under the random effects model, with a standardized mean difference as the effect size, the total effect size for the HNSCC subgroup was SMD = 0.52 [0.24; −0.79], with a test for heterogeneity of *p* = 0.20, *I* ^2^ = 28%, suggesting small heterogeneity. The total effect size for the HNSCC subgroup weighted 52.8% in the total data set available for inclusion in the analysis. The total effect size for all data sets that could be included in the study was SMD = 0.38 [0.14; 0.62], with a test for heterogeneity of *p* < 0.01, *I* ^2^ = 52%, suggesting large heterogeneity. (Due to image space limitations, this figure only captures the results of the analysis of the HNSCC subgroup and the analysis of the overall sample.) (D) Heatmap showing the differential expression of immune cell‐related genes (effector memory CD8 T cells, activated CD8 T cells, CD8+ T cells, B cells, activated B cells) between HPV‐positive and HPV‐negative samples in HNSCC_GSE72536. (E) Bar plot showing the enrichment of immune response‐related pathways in HNSCC_GSE72536 based on GSEA enrichment analysis. (F) Correlation coefficient matrix heatmap showing the correlation of *CDKN2A* with effector memory CD8 T‐cell‐related genes. *p < 0.05; **p < 0.01; ***p < 0.001. (G) Scatter plot showing the correlation between *CDKN2A* and the effector memory CD8 T‐cell‐related gene FLT3LG based on Spearman's correlation analysis. (H) Heatmap showing tumor‐associated pathways that differ significantly between HPV‐positive and HPV‐negative samples based on ssGSEA enrichment analysis (I) Scatter plot showing the correlation between *CDKN2A* and the ssGSEA score of the tumor‐associated pathway WP G1 TO S CELL CYCLE CONTROL based on Spearman's correlation analysis. (J) The forest plot shows the correlation between *CDKN2A* expression and the ssGSEA scores of WP WNT SIGNALING in HPV‐positive samples. Under the random effects model, with Fisher's *z*‐transformed correlation (ZCOR) as the effect size, the total effect size for the HNSCC subgroup was ZCOR = −0.34 [−0.48; −0.20] with a test for heterogeneity of *p* = 0.73, *I* ^2^ = 0%, suggesting small heterogeneity. The total effect size for the HNSCC subgroup weighted 24.6%. The total effect size for all data sets that could be included in the study was SMD = −0.24 [−0.37; −0.11], with a test for heterogeneity of *p* < 0.01, *I* ^2^ = 78%, suggesting large heterogeneity. (Due to image space limitations, this figure only captures the results of the analysis of the HNSCC subgroup and the analysis of the overall sample). HNSCC, head and neck squamous cell carcinomas; HPV, Human papillomavirus.

We found that multiple significantly upregulated genes (HPV positive vs. HPV negative, log_2_FC > 1, *p* < 0.05) were repeated in the data sets (Figure 6A). Among these genes, *CDKN2A* was significantly upregulated in 13 data sets. *CDKN2A/p16* has been reported to be found inactivated in a wide variety of solid tumors and more than 80% of oropharyngeal squamous cell carcinoma (OPSCC) [29, 30]. However, the impact of *CDKN2A* on the TIME of HPV‐associated tumors is currently unclear. Here, we made full use of HPVTIMER to address this question.

We performed a multimodule analysis using one of the HNSCC data set, HNSCC_GSE72536. We found significant differences in the immune infiltration scores of CD8+ T cells (quanTIseq: *p* < 0.05; EPIC: *p* < 0.0001) between HPV‐positive and HPV‐negative samples (Figure 6B, Figure S4A). A meta‐analysis showed that CD8+ T‐cell immune infiltration scores obtained by the quanTIseq algorithm differed significantly between HPV‐positive and HPV‐negative samples in all HNSCC samples (SMD = 0.52 [0.24; −0.79], *p* = 0.20, *I* ^2^ = 28%) and that CD8+ T‐cell infiltration was more significant in HPV‐positive samples (Figure 6C). The results were supported by DE analysis of immune cell‐associated genes and enrichment analysis of immune‐associated pathways. In the HNSCC_GSE72536 data set, genes associated with CD8+ T‐cell were significantly differentially expressed (*p* < 0.05, |log_2_FC| > 1.0) (Figure 6D). In addition, pathway enrichment analysis showed that multiple CD8+ T cell‐related pathways (CD8+ alpha‐beta T‐cell activation, etc.) were significantly enriched (*p*.adjust < 0.01) in HNSCC_GSE72536 (Figure 6E). To investigate the role of the target gene *CDKN2A* in the TIME, a correlation analysis was performed to explore the correlation between *CDKN2A* and CD8+ T cells. Spearman's correlation analysis revealed a significant positive correlation between *CDKN2A* and genes related to effector memory CD8+ T cells (Figure 6F, Figure S4B), such as *FLT3LG* (*r* = 0.72, *p* = 0.00015), *CCR5* (*r* = 0.71, *p* = 0.00016) (Figure 6G, Figure S4C–F). Also, *CDKN2A* showed a significant positive correlation with the immune infiltration scores of CD8+ T cells (EPIC: *r* = 0.8, *p* = 7.8e–06; quanTIseq: *r* = 0.57, *p* = 0.0045) (Figure S4G,H). According to the aforementioned evidence, *CDKN2A* may play a role in modulating CD8+ T cells to affect the TIME of HPV+HNSCC.

Further evidence suggests that *CDKN2A* may also regulate typical tumor pathways, such as the cell cycle and DNA damage, in addition to its role in immune‐related responses. We found that in HNSCC_GSE72536, the ssGSEA scores of the cell cycle‐related pathways (WP CELL CYCLE, etc.) and the DNA damage response pathway (e.g., WP DNA DAMAGE RESPONSE) were significantly upregulated in HPV‐positive samples (*p* < 0.05), whereas the ssGSEA scores of the Wnt/β‐catenin‐related pathways (WNT SIGNALING, etc.) were significantly downregulated in HPV‐positive samples (*p* < 0.05) (Figure 6H). According to a Spearman's correlation analysis, *CDKN2A* was significantly positively correlated with cell cycle‐related pathways (e.g., WP G1 TO S CELL CYCLE CONTROL [*r* = 0.55, *p* = 0.0069]), but significantly negatively correlated with Wnt/β‐catenin‐related pathways (e.g., WNT SIGNALING [*r* = −0.59, *p* = 0.0038]) (Figure 6I, Figure S5A–F). A Correlation meta‐analysis showed that *CDKN2A* was negatively correlated with the ssGSEA score of WP WNT SIGNALING (ZCOR = −0.34 [−0.48; −0.20], *p* = 0.73, *I* ^2^ = 0%) in HPV+HNSCC samples with less heterogeneity (Figure 6J). Thus, *CDKN2A* may be involved in regulating HPV+HNSCC through the cell cycle pathway, Wnt/b‐catenin pathway, and DNA damage response pathway.

## DISCUSSION

TIME is an important factor influencing the occurrence and development of HPV‐related tumors [3, 4, 5], and there exists a great potential for clinical research. However, the current studies of TIME in HPV‐associated tumors cannot meet the growing demand for diagnosis and treatment and are mostly limited by the sample size and sample type, and the applicability of their findings is restricted by the geography, ethnicity, and cancer species. Therefore, the development of the HPVTIMER platform for preliminary data mining becomes particularly urgent, meaningful, and efficient in this new format. HPVTIMER is a comprehensive TIME‐related online analysis site based on the GEO database that contains 7 immune infiltration algorithms, 8 cancer types, 60 immune cell types, 65 transcriptomic data sets of 2290 samples, and expression data from over 10,000 genes.

HPVTIMER is a powerful research tool designed to help researchers and clinicians analyze the TIME of HPV‐associated cancers and offers a wealth of features including rich cancer types, stunning visualizations, user friendliness, cost‐free operation, and easy accessibility (Table 1). Unlike other web‐based tools (e.g., CCDB, HNCDB, HNdb, and OrCGDB) that focus on single cancer types (e.g., CC, HNSCC, and OPSCC), TME research tools (e.g., CancerImmunityQTL, TIP, and TIMER2.0), and viral research tools (e.g., VISDB [29]), HPVTIMER is specifically designed to analyze HPV‐related cancers and thus offers a specialized approach while avoiding the restriction of focusing on only one type of cancer. With four analysis modules and meta‐analysis, HPVTIMER offers a complete analysis process and supports cross‐sectional comparisons of data sets. In particular, the immune infiltration analysis module provides access to seven immune infiltration estimation algorithms (CIBERSORT, CIBERSORT absolute mode, MCP‐counter (or mMCP‐counter for mice), EPIC, xCell, quanTIseq, and TIMER), which enables cross‐sectional and longitudinal analyses. Additionally, HPVTIMER provides visualization options, including 13 types of charts, and a user‐friendly interface that requires no prior computer programming knowledge. Furthermore, each submodule is equipped with a variety of visualization options, providing users with much flexibility. Moreover, access to HPVTIMER is straightforward and available without registration or login, which makes it a cost‐effective solution for researchers and clinicians.

**Table 1:** Comparison of HPVTIMER with other web tools.

	HPVTIMER	CancerImmunityQTL	TIMER2.0	TIP	VISDB	CCDB	HNCDB	HNdb	OrCGDB
Object of study	HPV‐associated tumor samples in GEO database	Cancer samples in TCGA database	Cancer samples in TCGA database	Cancer samples in TCGA database	Virus	CC samples	HNSCC samples in TCGA database and GEO database	HNSCC samples in TCGA database, GEO database and so on	OPSCC samples
Performing immune infiltration analysis	Yes	Yes	Yes	Yes	No	No	No	No	No
The number of immune infiltration algorithms	7	1	6	1	NA	NA	NA	NA	NA
Performing differential expression analysis	Yes	Yes	Yes	No	Yes	No	Yes	Yes	No
Performing correlation analysis	Yes	No	Yes	No	No	No	Yes	No	No
Performing pathway analysis	Yes	No	No	No	Yes	No	No	Yes	No
Performing comparison among data sets	Yes	Yes	Yes	Yes	Yes	No	YES	No	No
Performing meta‐analysis	Yes	No	No	No	No	No	No	No	No
The number of graphs offered to download	11	3	5	8	0	0	4	Unknown	0
Citation	\	[19]	[17]	[18]	[29]	[24]	[20]	[22]	[23]

Abbreviations: CC, cervical carcinoma; GEO, Gene Expression Omnibus; HNSCC, head and neck squamous cell carcinoma; HPV, human papillomavirus; OPSCC, oropharyngeal squamous cell carcinoma; TCGA, The Cancer Genome Atlas.

To further explain HPVTIMER, we show users an example of exploring potential HPV+HNSCC indicators, examining the effect of the marker on the TIME of HPV+HNSCC tumors, and investigating tumor‐associated pathways. We found that the potential biological signature of *CDKN2A* in HPV+HNSCC was closely related to the infiltration scores of CD8+T cells. Moreover, this signature could regulate HPV+HNSCC by modulating cell cycle‐related pathways. The results of the above example are consistent with previous reports [30, 31, 32]. As a result of HPV integration, the viral oncoproteins E6 and E7 are expressed, and these have the potential to inactivate the powerful tumor suppressor gene *pRb* and significantly increase the production of p16. Clinically, p16 (+) tumors have the same traits clinically as HPV (+) cancers, but p16 overexpression is not dependent on HPV [32, 33]. While *CDKN2A*, a known HNSCC gene [31], is involved in encoding the tumor suppressor protein p16, and p16 blocks the progression of the cell cycle from G1 to S phase by preventing the phosphorylation of Rb proteins [32]. This example is a good demonstration that helps HPVTIMER users fully understand the various modules of HPVTIMER.

Importantly, HPVTIEMR is now concentrating on addressing the effect of the HPV infection status on the TIME. Therefore, the discussion of HPV infection subtypes and HPV *E6/E7* genes is not currently covered. However, research on the transcriptional gene pool of HPV‐associated malignancies may not always be completely compatible with visual validation due to the diversity of HPV subtypes. Although immunotherapy has recently become more popular, most HNSCC tumors remain resistant to immune checkpoint blockade, and the precise mechanisms are still being researched [1, 2, 3]. As a result, the choices for treating HPV‐associated cancers have increased. Currently, HPVTIMER does not include a module dedicated to investigating the role of PD‐1/PD‐L1 in HPV‐associated malignancies.

Despite its various advantages, HPVTIMER still has some limitations. With regard to data, although HPVTIMER encompasses data from eight different cancer types, the sample sizes for small cell lung cancer (27), squamous skin cancer (28), papillary adenocarcinoma (29), retinoblastoma (30), and vulvar squamous carcinoma (31) are very limited. Additionally, the average sample size is small, which may result in a skewed distribution of potential data errors [34]. Moreover, because HPVTIMER is based on retrospective studies, the data exhibits a batch effect [35], and users thus need to be cautious when making cross‐data set comparisons. In terms of content in immune infiltration analysis, the level of infiltration of immune cells in HPV‐associated tumors may be influenced by multiple factors, such as the primary location, tumor type, TNM stage, and morphological heterogeneity [1, 4], and currently, HPVTIMER only explores the effect of tumor type on the TIME. In addition, HPVTIMER involves cross‐sectional analyses limited to the same species and does not perform joint analyses with multispecies data sets.

We will continue to incorporate HPV‐associated tumor data that meet the screening criteria within the GEO database and develop new features as needed. We anticipate that HPVTIMER will make it easier for users to explore the TIME of HPV‐related cancers and assist in improving patient diagnosis, staging, and immune‐targeted therapy.

## CONCLUSION

In this article, we present a web tool named HPVTIMER for analyzing the TIME of HPV‐associated cancers based on the GEO database, which involves 60 immune cell types, 65 transcriptome data sets, 2290 samples, and over 10,000 genes. HPVTIMER allows users to (i) explore the TIME of eight types of HPV‐associated cancers using seven immune infiltration estimation algorithms, (ii) longitudinally and systematically discover the impact of HPV infection on the biological characteristics of the eight HPV‐associated cancers, and (iii) compare data sets and find differences in the TIME of HPV‐associated cancers at different primary sites with different HPV infection states horizontally. This tool is a full‐featured and user‐friendly web‐based tool. We demonstrated an example to show users how to make use of HPVTIMER to explore what they are interested in. Our main goal was to provide researchers with a simple but efficient visualization tool with a high degree of freedom for studying the role of the TIME in the development of HPV‐associated cancers. We believe that HPVTIMER will prove to be a valuable resource for researchers and will help advance our understanding of HPV‐associated cancers and the role of the TIME in cancer development, which would ultimately lead to the discovery of new targets for tumor immunotherapy and improved treatments.

## MATERIALS AND METHODS

### Implementation

Users can freely conduct all analyses on the HPVTIMER website (http://www.hpvtimer.com/) without registration or login. HPVTIMER was constructed using the R package shiny [36], whereas server‐side and interactive data processing was performed with an R script (version 4.0.2, https://www.r-project.org/). HPVTIMER utilizes several third‐party tools, such as limma [37], meta [38], IOBR [39], clusterProfiler [40], and Pathview [41] (Table S3). The HPVTIMER website provides users with four analysis modules, namely, DE analysis, correlation analysis, immune infiltration analysis, and pathway analysis modules. The HPVTIMER website uses the DT package (https://rstudio.github.io/DT/shiny.html) to display the results and encourages users to query, select and download the data of interest freely. All graphs can be downloaded in Portable Document Format (PDF), Portable Network Graphics (PNG), or Scalable Vector Graphics (SVG) format.

### Data collection

We collected the transcriptomic data for all HPV‐associated cancers in the NCBI GEO database [42, 43] up to December 23, 2022. Only the data sets of HPV‐associated cancers with a definite HPV status (positive/negative) were included. We excluded healthy tissue samples and HPV‐positive or HPV‐negative tumor samples with a sample size of less than three in a given data set. Ultimately, we collected 65 data sets (Table S2) involving two species (human and mouse), three sample types (tumor tissues, cancer cell lines, and peripheral blood samples), and eight tumor types (CC, OPSCC, HNSCC, skin squamous cell carcinoma, digital papillary adenocarcinoma, retinoblastoma, small cell lung cancer, and vulvar squamous cell carcinoma).

### Data preprocessing

We preprocessed the data sets before analyzing each module.

#### High‐throughput sequencing

If the the source data are raw count data, we used the filterByExpr function [44] in the edgeR package to filter the count matrix for low‐expression genes. If the authors corrected the count matrix for quantification, we converted it to counts per million (CPM) or transcripts per million (TPM) format before filtering low‐expression genes. Only genes with gene expression (CPM/TPM) values greater than 0.5 in at least five samples were included in the subsequent analyses [44]. Ultimately, all RNA sequencing (RNA‐seq) data were uniformly converted to log_2_ (TPM+1) after data cleaning.

#### Single‐channel Agilent arrays

We used the limma package to read and preprocess the raw data acquired from single‐channel Agilent microarrays. Background correction was performed by limma‐backgroundCorrect with the method = “normexp” option. Between‐group variation was corrected by limma‐normalizeBetweenArrays with the method = “quantile” option. We only retained the probes with signal intensities above the background probes in at least half of the samples. We then confirmed whether the expression matrix was normalized and normalized it if it was not.

#### Affymetrix arrays

We used the affy package [45] to read and preprocess the raw data acquired from the HGU‐95 series and HGU‐133 series and the oligo package [46] for other series. The expression matrix was then normalized to RMA data first. The Genefilter‐nsFilter with “var.cutoff = 0.2” option was used to filter the low‐expression probes. We subsequently confirmed whether the expression matrix was normalized and normalized it if not.

#### Illumina BeadChips

We used the lumi package [47, 48, 49, 50] to read and preprocess the raw data. Only probes with “Detection *p* < 0.05” in at least half of the arrays were retained [37]. Subsequently, we confirmed whether the expression matrix was normalized and normalized it if it was not.

#### Two‐color microarrays

Raw data acquired from chips such as the two‐channel Agilent series chip, MWG Human 10k Array A, and Human Unigene3.1 cDNA Array 37.5K v1.0 were classified according to the image analysis program. We then read and preprocessed the data by limma‐read.maimages. Background correction was conducted by limma‐backgroundCorrect with the method = “normexp” option. The within‐group variation was corrected by limma‐normalizeWithinArrays with the method = “loess” option. The between‐group variation was corrected by limma‐normalizeBetweenArrays with the method = “quantile” option. The unknown genes or genes with a log_2_ (M) of 0 were filtered. We chose the maximum value of |log_2_ (M)| for duplicate genes.

### Functionalities

#### DE analysis

For RNA‐seq data sets, raw read counts were normalized using the trimmed mean of *M* values (TMM) in edgeR [44] to adjust the samples for differences in library size, and limma‐voom was used to transform these values into log_2_ CPM values using the default parameters. For microarray data and RNA‐seq data that were corrected and quantified, the preprocessed normalized data were used directly for DE analysis with the limma package. The visualization of the results of DE analysis involves two types of *p* values. One of them was calculated by the *t* test in the limma package, which was used to identify differentially expressed genes. The other was calculated by the Wilcoxon–Mann–Whitney test, which was used to determine whether to include a symbol indicating significance symbol in the graphs.

#### Correlation analysis

We conducted the correlation analysis using the Pearson and Spearman methods. We collected a total of 17,970 genes associated with hallmark gene sets, canonical pathways and ontology gene sets from the MSigDB [28] and calculated enrichment scores for each pathway using GSVA‐gsva with the method = “ssgsea” option. These enrichment scores will be used for correlation exploration within the “Scatter plot” submodule. Unfortunately, the ssGSEA algorithm does not work for two‐color array data; thus, the ssGSEA enrichment scores could not be calculated for two‐color data sets. Using the “metacor” function in the meta package [38], we conducted a meta‐analysis of correlation coefficients among gene expressions/pathway' ssGSEA scores/immune infiltration scores.

#### Immune infiltration

We used seven immune infiltration estimation algorithms (CIBERSORT [49], CIBERSORT absolute mode [51], MCP‐counter (or mMCP‐counter for mice) [52, 53], EPIC [54], xCell [55], quanTIseq [56], and TIMER [57]) for immune infiltration analysis. Due to the distinct principles, the methods that can be used for intrasample comparisons between cell types are CIBERSORT, CIBERSORT absolute mode, EPIC, and quanTIseq, and the methods that can be used for intersample comparisons between the same cell types are CIBERSORT absolute mode, MCP‐counter (or mMCP‐counter for mice), EPIC, quanTIseq, TIMER, and xCell [58]. For mouse data sets, with the exception of the mMCP‐counter algorithm used to calculate the mouse gene expression matrix, we first used the biomaRt package [59, 60] to map mouse genes to human genes before using other algorithms to perform immune infiltration analysis. Of note, not all immune infiltration estimation algorithms are applicable to each data set; for example, the xCell algorithm does not work for two‐color array data, and the TIMER algorithm is only applicable to cervical cancer and HNSCC samples. Using the “metacont” function in the meta package [38], we conducted a meta‐analysis of immune cell immune infiltration scores to investigate their variation across HPV‐positive and HPV‐negative samples.

#### Pathway analysis

We used two algorithms, ORA and GSEA, from the clusterProfiler package [40, 61] to conduct enrichment analysis based on gene ontology (GO) terms, KEGG pathways, and some MSigDB gene sets (hallmark gene sets, ontology gene sets, and canonical pathways) (using the default parameter settings, i.e., “minGSSize = 10, maxGSSize = 500”). For the GSEA algorithm, we included all genes in the selected data set, whereas only the significantly differentially expressed genes (*p* < 0.05 and |log_2_FC| > 1.0) in the selected data set were included for the ORA algorithm. Both algorithms filter significant pathways based on *p*.adjust < 0.05 [40, 61].

#### Data

Thirty of the 65 data sets built into HPVTIMER have clinical characteristics information available in public databases. We presented this information using the DT package. We used the “CreateTableOne” function in the tableone package [62] to test for variations in clinical traits based on the HPV infection status.

### Candidate gene screening

We will utilize an example based on the integrated data sets to help users better navigate HPVTIMER.

Genes that were significantly differentially expressed in more than half of the embedded data sets (|log_2_FC| > 1, *p* < 0.05) were chosen as candidate markers using the “DE analysis‐overall” module. Immune infiltration analysis of the target data set was performed using the “Immune infiltration” module to screen for immune cells with significantly different levels of immune infiltration between HPV‐positive and HPV‐negative samples. Meta‐analysis was performed to identify immune cells that consistently differed in immune infiltration across all embedded HNSCC data sets. We further explored the differential expression of immune cell‐related genes and immune‐related pathway enrichment using the “DE analysis‐immune cell” module and the “Pathway analysis—Pathway Enrichment” module (based on the GSEA algorithm). To verify the involvement of the candidate marker in influencing the TIME, we used Spearman's correlation analysis provided by the “Correlation‐Scatter plot” module to explore the relationship between the candidate marker and immune cells, including the correlation between the candidate marker and immune cell‐associated gene expression and the correlation between the candidate marker and immune infiltration scores.

To further investigate the specific mechanism by which this candidate gene affects the TIME, we performed ssGSEA pathway enrichment analysis using the “Pathway analysis‐ssGSEA” module and used the U‐test to identify pathways that were significantly different between HPV‐positive and HPV‐negative samples. We explored the correlation between this candidate marker and the target pathway ssGSEA score using the Spearman's correlation analysis provided by the “Correlation‐Scatter plot” module. Subsequently, we used the “Correlation—Meta analysis” module to assess whether the findings of the above analysis were consistent across all relevant data sets.

## AUTHOR CONTRIBUTIONS

All authors contributed to the study's conception and design. Liying Liu and Yanan Xie were responsible for methodology, formal analysis, investigation, data curation, visualization, writing the original draft, and editing. Hong Yang, Anqi Lin, and Minjun Dong were responsible for methodology, investigation, data curation, review, editing, and project administration. Haitao Wang, Cangang Zhang, and Zaoqu Liu were responsible for conceptualization, methodology, validation, review, and supervision. Peng Luo, Shuofeng Yuan, Jian Zhang, and Quan Cheng were responsible for conceptualization, supervision, project administration, funding acquisition, review, and editing. All authors read and approved the final manuscript.

## CONFLICT OF INTEREST STATEMENT

The authors declare no conflict of interest.

## Supporting information

Supporting information.

Supporting information.

## Data Availability

The HPVTIMER website is available at http://www.hpvtimer.com/. The data sets analyzed in this study were derived from the Gene Expression Omnibus database (https://www.ncbi.nlm.nih.gov/gds). All other data generated and analyzed in this study are available from the corresponding authors upon reasonable request. Supporting Information (figures, tables, scripts, graphical abstract, slides, videos, Chinese translated version, and update materials) may be found in the online DOI or iMeta Science http://www.imeta.science/. The HPVTIMER website is available at http://www.hpvtimer.com/. The code for building the website is available at https://github.com/SMULiuLY/HPVTIMER/. Further information and requests should be directed to and will be fulfilled by the corresponding authors. Supporting Information (figures, tables, scripts, graphical abstract, slides, videos, Chinese translated version, and updated materials) may be found in the online DOI or iMeta Science http://www.imeta.science/.
