# Supplementary material for: HPVTIMER: A shiny web application for tumor immune estimation in human papillomavirus‐associated cancers
Source: Imeta. 2023 Aug 12;2(3):e130. doi: 10.1002/imt2.130 (PMC10989930; doi:10.1002/imt2.130)
Supplement: Supplementary file 1 — Supporting information. [file IMT2-2-e130-s001.docx]

**HPVTIMER: A Shiny web application for tumor immune estimation in human papillomavirus-associated cancers**

**Running title: HPVTIMER**

Liying Liu^1,2†^, Yanan Xie^1,3†^, Hong Yang^1†^, Anqi Lin^1†^, Minjun Dong^4†^, Haitao Wang^5^, Cangang Zhang^6^, Zaoqu Liu^7^, Quan Cheng^8,9*^, Jian Zhang^1*^, Shuofeng Yuan^10,11*^, Peng Luo^1*^

^1^ Department of Oncology, Zhujiang Hospital, Southern Medical University, Guangzhou, 510282, Guangdong, China

^2^ The First Clinical Medical School, Southern Medical University, Guangzhou, 510515, Guangdong, China

^3^ The Second Clinical Medicine School, Southern Medical University, Guangzhou, 510515, Guangdong, China

^4^ Department of Surgical Oncology, Sir Run Run Shaw Hospital affiliated to Zhejiang University, School of Medicine. Hang Zhou, China

^5^ Thoracic Surgery Branch, Center for Cancer Research, National Institutes of Health, Bethesda, MD, USA

^6^ Department of Pathogenic Microbiology and Immunology, School of Basic Medical Sciences, Xi'an Jiaotong University, Xi'an, Shaanxi, China

^7^ Department of Interventional Radiology, The First Affiliated Hospital of Zhengzhou University, Zhengzhou, Henan, China

^8^ Department of Neurosurgery, Xiangya Hospital, Central South University, Changsha, 410008, Hunan, China Xiangya Hospital, Central South University, Hunan, China

^9^ National Clinical Research Center for Geriatric Disorders, Xiangya Hospital, Central South University, Hunan, China

^10^ Department of Infectious Disease and Microbiology, The University of Hong Kong-Shenzhen Hospital, Shenzhen, 518009, China

^11^ State Key Laboratory of Emerging Infectious Diseases, Carol Yu Centre for Infection, Department of Microbiology, School of Clinical Medicine, Li Ka Shing Faculty of Medicine, The University of Hong Kong, Hong Kong, China

^†^ These authors contributed equally to this work and share the first authorship.

^*^ Corresponding authors: Peng Luo (luopeng@smu.edu.cn), Shuofeng Yuan (yuansf@hku.hk), Jian Zhang (zhangjian@i.smu.edu.cn) and Quan Cheng (chengquan@csu.edu.cn).

Figure S1. Box plot of the expression of genes of interest related to immune cells identified by differential expression analysis. The Wilcoxon–Mann–Whitney test was used to compare the expression of the genes of interest between HPV-positive and HPV-negative samples. ns: *p* > 0.05; * *p* < 0.05; ** *p* < 0.01; *** *p* < 0.001; **** *p* < 0.0001.

Figure S2. Examples of visualizations of pathway analysis results. (A) The pathway enrichment analysis reveals the upregulation and downregulation of specific pathways in bar plots. (B) Heatmap of ssGSEA scores of pathways of interest from the dataset. The Wilcoxon–Mann–Whitney test was used to compare whether the ssGSEA scores of pathways differed between HPV-positive and HPV-negative samples.

Figure S3. (A) Example of data presentation. The dataset summary table presents specific information about the 65 datasets embedded in HPVTIMER. (B) The immune cell-associated gene table presents the list of immune cell-related genes used in the “DE analysis-Immune cells” module.

Figure S4. (A) Immune infiltration levels (based on the EPIC algorithm) were compared between HPV-positive and HPV-negative samples in HNSCC_GSE72536 using the Wilcoxon–Mann–Whitney test. ns: *p* > 0.05; * *p* < 0.05; ** *p* < 0.01; *** *p* < 0.001; **** *p* < 0.0001. (B) Correlation coefficient heatmap showing the correlation of *CDKN2A* with effector memory CD8 T-cell-related genes. (C-F) Scatter plot of the correlation between *CDKN2A* and other effector memory CD8 T-cell-related genes (CCR5, LIME1, ACAP1, GZMM) based on Spearman correlation analysis. (G-K) Scatter plot of the correlation between *CDKN2A* and immune infiltration scores (based on the quanTIseq algorithm and EPIC algorithm) of CD8+ T cells based on Spearman correlation analysis.

Figure S5. Scatter plot of the correlation between *CDKN2A* and ssGSEA scores of the cell cycle-related pathways (BIOCARTA CELLCYCLE PATHWAY (A), and GOBP CELL CYCLE ARREST (B)), DNA damage response pathway (WP DNA DAMAGE RESPONSE) (C), Wnt/β-catenin-related pathways (WNT SIGNALING (D), BIOCARTA WNT PATHWAY (E), and KEGG WNT SIGNALING PATHWAY (F)) based on Spearman correlation analysis.
